# Impact of animal socioecology on gut microbial communities: Insights from wild meerkats in the Kalahari

**DOI:** 10.1111/1365-2656.70168

**Published:** 2025-10-30

**Authors:** Krishna Balasubramaniam, Nadine Mueller‐Klein, Tim Vink, Timothy H. Clutton‐Brock, Marta B. Manser, Simone Sommer

**Affiliations:** ^1^ School of Life Sciences, Faculty of Science & Engineering, Anglia Ruskin University Cambridge UK; ^2^ Institute for Evolutionary Ecology and Conservation Genomics Ulm University Ulm Germany; ^3^ Kalahari Research Trust Van Zylsrus South Africa; ^4^ Large Animal Research Group, Department of Zoology University of Cambridge Cambridge UK; ^5^ Mammal Research Institute, University of Pretoria Pretoria South Africa; ^6^ Department of Evolutionary Biology and Environmental Studies University of Zurich Zurich Switzerland

**Keywords:** animal socioecology, gut microbial communities, joint‐species‐distribution model (JSDM), meerkats (*Suricata suricatta*), microbial co‐occurrence networks, social microbiome

## Abstract

1. The social organisation of animals likely shapes the composition, diversity and stability of microbiomes, giving rise to the concept of the ‘social microbiome’—microbial communities shared within and across social units, or ‘islands’, ranging from individuals to entire ecosystems. Understanding the connections and their underlying drivers is crucial for revealing how socioecology influences microbiomes and associated health outcomes. However, empirical assessments are still limited, and the relative influence of social organisation compared to intrinsic (biological) and extrinsic (environmental) factors in shaping microbiomes is particularly unclear.
2. Here, we used a long‐term, individual‐based study of Kalahari meerkats (*Suricata suricatta*) to test predictions from the social microbiome concept. We assessed the relative influence of social factors, biological traits and environmental variables on gut microbial communities, while also accounting for the effects of microbial phylogenetic relatedness and within‐host associations or co‐occurrence independent of phylogeny.
3. Meerkat microbiomes exhibited highly ‘nested’ and weakly ‘modular’ structures: individuals with lower diversity hosted amplicon sequence variants (ASVs) that were subsets of the overall community, though some bacterial taxa clustered distinctly among hosts. Microbiomes were more similar within social groups than between them.
4. Group membership strongly influenced the co‐occurrence of many beneficial ASVs, as well as a few potentially harmful ones. This effect was stronger than that of kinship, though closer relatives shared more similar microbiomes within some groups. While a range of social, biological and environmental factors influenced bacterial abundance, group membership, individual age and sampling time since sunrise had the most significant impact. ASV‐ASV co‐occurrence within hosts, independent of phylogeny, also played a major role. In contrast, individual‐level social traits (e.g. dominance, immigration), other environmental (e.g. sampling temperature, rainfall, hours since foraging), demographic (sex) and health‐related factors (body condition, disease status) had weaker effects on bacterial abundance.
5. We show that gut microbiomes are shaped by a combination of factors, highlighting the importance of separating the effects of social organisation from individual social traits, biological factors, environmental influences and microbe–microbe interactions. By identifying drivers of both beneficial and detrimental bacterial co‐occurrence, we provide a foundation for assessing how the social microbiome affects animal health and fitness.

## Graphical abstract

Our study addresses critical gaps at the nexus of animal and microbial ecologies, adding much‐needed empirical support to recent theoretical frameworks that have conceptualised gut microbiomes as being strongly influenced by animal sociality and group‐living, and bringing benefits that may have strongly (and reciprocally) influenced the evolution of social life.

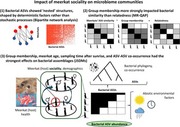

## 1 INTRODUCTION

Animal social organisation, such as group living, spatial proximity and behavioural interactions with conspecifics, can strongly influence their gut microbiomes (Archie & Tung, 2015; Baniel & Charpentier, 2024; Sarkar et al., 2020). Understanding the ecological and evolutionary drivers of microbiome composition is important, as microbiomes play essential roles in shaping key aspects of animal behaviour, health and fitness in complex and multifaceted ways (Libertucci & Young, 2018; Lombardo, 2008; Nunn et al., 2015). Likewise, host behavioural biology, in particular sociality and group living, can reciprocally influence the acquisition and transmission of microbiota (Archie & Tung, 2015; Nunn et al., 2015; Sarkar et al., 2020). Over the past 15 years, studies across diverse wildlife taxa have reported associations between animal social organisation and the bacterial diversity and composition of microbiomes. Examples include bumblebees (*Bombus bombini*: Koch & Schmid‐Hempel, 2011), several African antelopes (VanderWaal et al., 2014), Cape buffalo (*Syncerus caffer*, Couch et al., 2021), spotted hyenas (*Crocuta crocuta*, Rojas et al., 2023), Antarctic fur seals (*Arctocephalus gazella*, Grosser et al., 2019), yellow‐bellied marmots (*Marmota flaviventer*: Pfau et al., 2023) and several non‐human primates such as Verreaux's sifakas (*Propithecus verreauxi*, Perofsky et al., 2017), black howler monkeys (*Alouatta pigra*, Amato et al., 2017), yellow baboons (*Papio cynocephalus*, Tung et al., 2015), rhesus macaques (*Macaca mulatta*: Johnson et al., 2022) and chimpanzees (*Pan troglodytes*, Degnan et al., 2012; Moeller et al., 2016). These findings have led to the concept of the ‘social microbiome,’ which, informed by metacommunity theory, describes the abundance, diversity and distribution of gut microbes within and across ‘islands’ or ‘archipelagos’ of nested host social units, ranging from individuals to groups, populations and entire ecosystems (Baniel & Charpentier, 2024; Sarkar et al., 2020, 2024).

Despite growing interest, key gaps still remain in our understanding of the social microbiome. Different aspects of animal social organisation can influence microbial composition in complex ways, making it challenging to disentangle their relative effects. Empirical studies to date have predominantly focused on detecting (or inferring) evidence of direct contact or shared use of space as potential mechanisms driving microbial sharing or transmission (reviewed in Sarkar et al., 2020). However, broader aspects of animal social organisation, like individual roles within groups, group membership and kinship relationships—may drive and influence patterns of contact‐based microbial transmission (Archie & Tung, 2015; Sarkar et al., 2020; VanderWaal et al., 2014). According to the social microbiome framework (Sarkar et al., 2020), microbial sharing is expected to be more frequent among members of the same group or between closely related individuals, particularly in societies with high genetic relatedness such as eusocial insects (Nowak et al., 2010), cooperative breeders like meerkats (*Suricata suricatta*: Clutton‐Brock & Manser, 2016) and species with matrilineal kin structures that drive strong kin bias in affiliative social interactions, including spotted hyenas (Wahaj et al., 2004) and nonhuman primates like baboons and macaques (Silk, 2002). However, there is little empirical research on the relative impact of group membership and kinship on microbiomes. Beyond organisational effects, microbial acquisition can also be shaped by individual social behaviours and strategies, such as dispersal, immigration or dominance rank (Archie & Tung, 2015; Baniel & Charpentier, 2024; Sarkar et al., 2020). Yet, few studies have assessed the relative importance of organisational versus individual‐specific aspects of social organisation on the social microbiome.

Another key challenge is separating the effects of social factors from those of host‐specific biological traits and environmental factors. For instance, microbiomes can be strongly shaped by intrinsic biological characteristics of hosts, such as age (meerkats: Risely et al., 2021; Egyptian mongoose, *Herpestes ichneumon*: Pereira et al., 2020), sex (rodents: Org et al., 2016; passerine birds: Yan et al., 2022) and their infectious disease status (e.g. stages of tuberculosis infection among long‐tailed macaques, *Macaca fascicularis*: Sawaswong et al., 2024 and meerkats: Risely et al., 2023). Similarly, a growing body of research links shifts in microbiome composition to environmental factors. Gut microbial turnover can be driven by climate change (Risely et al., 2021), diurnal‐to‐nocturnal circadian rhythms (Risely et al., 2021), seasonality (Sun et al., 2016), spatio‐temporal variation in resource availability and resulting dietary changes (Ingala et al., 2019) and anthropogenic impacts (Fackelmann et al., 2021). However, the relative influence of social factors compared to individual biological traits and environmental variables in shaping microbiome composition remains unclear and underexplored.

Finally, most studies of the social microbiome have focused on characterising the composition of microbial communities between hosts, examining metrics like taxonomic richness, abundance and diversity. In contrast, much less is known about the structure of these communities, particularly patterns of microbe–microbe associations or ‘co‐occurrence’ within individual hosts (Balasubramaniam et al., 2025; Fountain‐Jones et al., 2019; Risely et al., 2023). Understanding microbial community structure and the influence of host social organisation on these patterns is crucial, as growing evidence suggests that microbial co‐occurrence in wildlife is not simply the result of random assembly (Anthony et al., 2015; Costello et al., 2012; Risely et al., 2023). Rather, these patterns are shaped by complex interactions that operate across multiple levels of host organisation, from individual animals to groups, populations and entire ecosystems (French & Holmes, 2020; Ladau & Eloe‐Fadrosh, 2019). For instance, highly abundant bacteria may be more widely shared across individual animal hosts, while rarer taxa tend to occur in only a few individuals, indicating a ‘nested’ pattern. In contrast, some bacterial taxa may follow a ‘modular’ or ‘segregated’ distribution, appearing more often within specific subsets of individuals. These patterns support the concept of social microbiomes as metacommunities shaped by the scales of social organisation in which they are embedded (Baniel & Charpentier, 2024; Sarkar et al., 2020).

Network‐based analytical approaches have been used to explore these gaps, enabling the analysis of associations between microbial taxonomic units and host entities—such as individuals or species—through bimodal (bipartite) ecological networks (Fountain‐Jones et al., 2019; Massol et al., 2021), or microbe–microbe co‐occurrence within hosts via unimodal networks (Anthony et al., 2015; Li et al., 2019; Riera & Baldo, 2020; Risely et al., 2023). However, classical network analyses are limited in their ability to quantify the relative importance of host, environmental and microbe‐specific factors on microbial community structure (Fountain‐Jones et al., 2019; Massol et al., 2021). Joint species distribution models (JSDMs), widely used in macroecology to assess environmental effects on species diversity and sympatry (Ovaskainen et al., 2017; Pollock et al., 2014; Tikhonov et al., 2020), have recently emerged as valuable tools to address this problem (Fountain‐Jones et al., 2019). These models capture co‐variation in the responses of individuals (or other taxonomic units) to features of their environment, considering taxon‐specific traits and phylogenetic relationships (Ovaskainen et al., 2017; Pollock et al., 2014; Tikhonov et al., 2020). Importantly, JSDMs can generate microbial co‐occurrence networks based on *residual* associations—co‐occurrences that remain after accounting for host‐ or sample‐specific variables and microbial phylogeny. This makes JSDMs powerful tools for analysing microbial co‐occurrence and advancing our understanding of metacommunity dynamics in host‐associated microbiomes. To date, they have been used to assess the co‐occurrence of endemic and epidemic pathogens in African lions (*Panthera leo*: Fountain‐Jones et al., 2019); gut microbiota interactions in Neotropical bird species (Björk et al., 2018); the co‐occurrence of pathogenic bacteria, helminths and protozoa in rodents (Mariën et al., 2022); and viral co‐occurrence and community structure in rhesus macaques (*Macaca mulatta*: Balasubramaniam et al., 2025).

In this study, we investigated the relative contributions of host social organisation, individual biological traits and environmental factors on gut microbial communities, using wild meerkats in the Kalahari as a model system. Meerkats are cooperatively breeding small carnivores that live in arid deserts in Southern Africa (Clutton‐Brock & Manser, 2016). Over three decades of research on the Kalahari meerkat population has yielded detailed insights into their social organisation. Meerkats live in small‐ to medium‐sized groups with generally high, but variable, relatedness among group members. Their social system includes female philopatry and male dispersal into new groups, and a clear division of social status, with dominant pairs suppressing reproduction in subordinate helpers (reviewed in Clutton‐Brock & Manser, 2016). Recent research has shown that various aspects of meerkat health, including their microbiota, are influenced by factors such as circadian (diurnal) rhythms (Risely et al., 2021), age (Risely et al., 2022), climate change (specifically shifts to warmer temperatures: Paniw et al., 2022; Risely et al., 2023) and infectious disease, notably their susceptibility to tuberculosis caused by the endemic pathogen *Mycobacterium suricattae* (Muller‐Klein et al., 2022; Paniw et al., 2022; Patterson et al., 2022; Risely et al., 2023). Here, we complement these longitudinal studies by focusing on shorter time frames and well‐sampled groups to examine the relative effects of social organisation (hereafter ‘social factors’) versus biological traits and exposure to asocial environmental factors (hereafter just ‘environmental factors’) on microbiome diversity and composition.

Specifically, we investigated whether the meerkat microbiota exhibit non‐random patterns in community structure—that is, whether they display nested structures, indicative of widespread bacterial abundance across many meerkats, or modular/segregated structures, suggesting greater host‐bacterial taxon specificity. In doing so, we aimed to establish a premise for expecting bacterial community structure to be shaped by deterministic factors rather than solely by stochastic processes (Hayashi et al., 2024; Zhou & Ning, 2017). Next, we examined whether meerkats' group membership or kinship influenced bacterial community composition—specifically, whether individuals from the same group (vs. different groups) or those with closer (vs. more distant) kin had more similar microbiota. We then applied JSDMs to evaluate the relative importance of meerkat social factors—such as group membership, kinship, immigration and social status—as well as individual biological traits (age, sex, body condition, disease status) and environmental variables (temperature, rainfall, time since foraging and sunrise) in shaping bacterial community composition. Additionally, we examined how bacterial phylogenetic relationships and the co‐occurrence of specific taxa, particularly those most strongly influenced by meerkat social organisation, contribute to the structure of the microbiota. Our study offers valuable insights into the factors governing microbial communities, with implications for understanding how social dynamics impact the resilience and health of animal populations.

## 2 MATERIALS AND METHODS

### 2.1 Ethics and diversity statement

The fieldwork component of this research was conducted with the permission/approval of The South African Northern Cape Department of Environment and Nature Conservation (FAUNA 1020/2016), the ethical committee of Pretoria University (Permit 384 number: EC031‐13) in South Africa and the local stakeholders of the Kuruman River Reserve, which is also the location of the Kalahari Meerkat Project (KMP) in South Africa. We are grateful to the contributions of numerous project volunteers from diverse backgrounds, including several local (South African born) as well as international volunteers, who routinely conduct fieldwork at the KMP.

### 2.2 Study population and faecal sample collection

We studied eight social groups of meerkats observed across three study periods: four groups in 2006–2007, two in 2014–2015 and two in 2016–2017 (Table S1). The groups are part of a large population of wild meerkats inhabiting the Kalahari semi‐desert region, spanning an area of 50–60 km^2^. The study area is situated within the boundaries of the Kuruman River Reserve and encompasses adjacent agricultural lands in South Africa (26.96° S, 21.83° E). The study population is subject to routine monitoring by members of the Kalahari Meerkat Project, who have been engaged in the systematic collection of demographic, behavioural, life history, environmental and health‐related datasets on multiple groups since 1993 (Clutton‐Brock & Manser, 2016). The selection of groups and data collection periods for this study was based on the highest proportion of animals from which gut microbial data, as well as sample‐specific metadata necessary for this study, were available (Risely et al., 2021, 2022, 2023). Faecal samples were collected directly from the ground once a meerkat was observed defaecating. The samples were stored in ice packs and frozen within 8 h of collection by either storing in a −20°C freezer on site or by freeze‐drying at room temperature. The latter method was adopted after 2008 since it proved to be more advantageous in terms of freeze‐dried DNA being more robust to long‐term storage and temperature changes (Bensch et al., 2022; Blekhman et al., 2016). Diagnostic tests conducted in a previous study confirmed that the storage method had, at best, a negligible effect on microbiota composition or stability (Risely et al., 2021).

### 2.3 DNA extraction and library preparation for high‐throughput sequencing of the gut microbial community

All faecal samples were treated with NAP buffer prior to DNA extraction. From these, a subsample of 0.6 ± 0.05 μg (wet) was extracted, to which 3 μL of ZymoBIOMICS Spike‐in Control I (High Microbial Load) was added as an internal standard. This consisted of cells belonging to *Imtechella halotolerans* and *Allobacillus halotolerans*, two thermophilic bacterial species that are rarely found in faecal microbiota communities. This allows the absolute abundance of each bacterial taxon after sequencing to be standardised to a known number of cells of uncommon bacterial taxa contained in the standard. Although this method estimates bacterial 16S copy number or (hereafter) sequence ‘read depth’ rather than absolute abundance, it is still highly correlated with absolute abundance given a high degree of standardisation of faecal sample mass (Hardwick et al., 2017; Lin et al., 2019; Tourlousse et al., 2017).

Genomic DNA was extracted using the NucleoSpin 96 Soil kit (Macherey‐Nagel) according to the manufacturer's instructions. We then used the predesigned primer pair of 515 F (5′‐GTGCCAGCMGCCGCGGTAA‐3′) and 806R (5′‐GGACTACHVGGGTWTCTAAT‐3′) to amplify the hypervariable V4 region of the 16S rRNA gene. The Fluidigm Access Array™ for Illumina Sequencing Systems was used for indexing and adding Illumina adaptor sequences. After purification (NucleoMag® NGS Clean‐up and Size Select; Macherey‐Nagel) and quantification (QuantiFlour® dsDNA System; Promega) of the barcoded samples, the normalised pooled sample library was sequenced as a paired‐end run on an Illumina MiSeq platform at the Institute of Evolutionary Ecology and Conservation Genomics, Ulm University, Germany. Samples were sequenced in four Illumina runs (MiSeq Reagent Kit v2, 500‐cycles). Extraction and PCR‐negative controls were included in all runs.

### 2.4 Bioinformatics and normalisation of microbiota sequencing data

All sequence reads were processed using QIIME2 version 2020.2. Sequences were merged, quality filtered and chimeras were removed using the DADA2 pipeline to generate bacterial amplicon sequence variants (hereafter ASVs) (Callahan et al., 2016, 2017). Primers were trimmed and reads were truncated at 244 (forward) and 235 (reverse) base pairs. Bacterial ASVs were assigned a taxonomy using SILVA version 132 (Pruesse et al., 2007). A tree was constructed using the fragment insertion method of QIIME2. ASVs were filtered if they were not bacteria, not assigned to a phylum (as these are assumed to be spurious), or if they were classified as mitochondria or chloroplasts. To eliminate sand‐derived microbes from the dataset, we applied the decontam::isContaminant function (Davis et al., 2018) using the ‘prevalence’ method, with 15 sand samples as a reference. Despite this approach, it is still likely that meerkats acquired microbes from the sand through behaviours such as movement, burrow use and foraging for invertebrate prey (Clutton‐Brock & Manser, 2016; Jubber et al., 2025; Strandburg‐Peshkin et al., 2020). To normalise the sequence read depth values of bacterial ASVs, we divided each value by the abundance values of our control taxa, after which we removed the control taxa from the dataset. As some samples had a very high relative abundance of spike‐in, we only retained samples for which the total microbiota read depth values were >5000.

In total, we analysed 528 faecal samples collected from 146 meerkat individuals across the eight groups (Table S1). From a total of 26,122 ASVs, we excluded rare taxa represented by less than 100 reads and then collapsed ASVs to the genus level, as we were primarily interested in bacterial community structure, similarity and co‐occurrence at this level. Thus, all data analyses were performed at the genus level on the presence–absence and/or read depth values of a final list of 119 bacterial ASVs that were prevalent in at least 10% of the samples across all three time periods (Table S2). Applying this 10% prevalence criteria *within* periods, we included and analysed 108 unique ASVs in period I, 95 in period II and 102 in period III, with a total of 83 ASVs from this list being considered to be the most prevalent (in ≥10% in all study periods) both across the entire dataset as well as within each period (Table S2).

### 2.5 Sample metadata on social, biological and environmental variables

For each sample and associated meerkat ID, we extracted and analysed metadata on aspects of meerkat social organisation, individual‐specific biological factors and exposure to environmental variables from the Kalahari Meerkat Project (KMP) long‐term database. Table 1 provides details of each covariate (sub)category and detailed definitions.

**TABLE 1:** Details of meerkat social, biological and environmental factors included as covariates in the study.

Covariate category (subcategory)	Covariate	Definition
Social	Group membership	Group assignment of meerkats at the time of sampling, i.e. being part of a continuous association of two or more individuals that includes animals of different age/sex classes (Clutton‐Brock & Manser, 2016)
Social	Kinship	Degree of maternal relatedness between pairs of meerkats, estimated as ‘Wang's coefficient of relatedness’ from a multigenerational pedigree based on parentage established by genetic analysis of 18 microsatellite markers, supplemented by observational field data on maternity where genetic data were missing (Nielsen et al., 2012)
Social	Recent immigration into the group	Whether (or not) the meerkat individual had recently immigrated to the group in which it was sampled, i.e. within ≤3 months prior to sampling (Clutton‐Brock & Manser, 2016)
Social	Dominance status	Categorisation of an individual as either ‘dominant’ (based on predefined behaviours such as regular substrate or group member marking, aggressive interactions with conspecifics, mate‐guarding and/or being a member of the primary breeding pair at the time of sampling), or ‘subordinate’ (all other individuals) (Clutton‐Brock & Manser, 2016)
Host biological (demographic)	Age	Age of an individual (in years) at the time of sampling
Host biological (demographic)	Sex	Sex of an individual (categorical: male vs. female)
Host biological (health‐related)	Body condition	Residual body condition or body mass of an individual at the time of sampling. Values were obtained from the residuals of a model regressing body weight on the age and the time of day (categorical variable of morning versus evening, since meerkats are typically weighed twice on the same day) (Risely et al., 2022, 2023)
Host biological (health‐related)	Disease (Tb infection) status	Meerkats are susceptible to tuberculosis, caused by the endemic bacterium *Mycobacterium suricattae*, which almost always leads to symptomatic disease (submandibular swelling) and death after a long and unpredictable latent phase (reviewed in Muller‐Klein et al., 2022). In line with previous research (Risely et al., 2023), each meerkat was therefore classified into one of three Tb categories at the time of sampling: ‘unexposed’: never cohabited or been a part of a group with one or more Tb symptomatic or deceased individuals; ‘exposed_asymptomatic’: cohabited with a diseased individual but had never shown active Tb until the date of sampling; ‘exposed_symptomatic’: showed active signs of Tb at the time of sampling
Environmental (climatic)	Temperature	Prevailing temperature (°C) at the time of sampling, which previous research has shown to have a strong influence on the temporal turnover of meerkat microbiome composition (Risely et al., 2021)
Environmental (climatic)	Rainfall	Average rainfall (in mm) during the month prior to sample collection (Risely et al., 2021)
Environmental (temporal)	Time since foraging	Hours elapsed since the start of the last morning or afternoon foraging session, which previous research has shown to have a strong influence on the temporal turnover of meerkat microbiome composition (Risely et al., 2022, 2023)
Environmental (temporal)	Hours after sunrise	Hours elapsed since sunrise on the day of sampling, at which the sample was collected, which previous research has shown to have a strong influence on the temporal turnover of meerkat microbiome composition (Risely et al., 2022, 2023)

### 2.6 Microbial network analysis and statistical assessments

#### 2.6.1 Do meerkat microbiota exhibit non‐random patterns in community structure?

We used a bipartite network analysis to investigate bacterial community structure, that is, whether bacterial ASVs among meerkats exhibit non‐random patterns of nestedness, modularity or segregation among hosts (Figure S1: adapted from Strona & Veech, 2015). In metacommunity ecology, ‘nestedness’ describes a pattern where sites with lower species richness are subsets of sites with higher species richness, creating a gradient of diversity. When applied to microbial communities within individual meerkats, which we consider as ‘islands’, we asked whether their bacterial communities followed a similar gradient of species richness. Specifically, we sought to determine if meerkats with lower microbial diversity hosted bacterial communities that were a subset of those found in meerkats with higher diversity (Strona & Veech, 2015). Alternatively, we considered whether the microbial communities exhibited ‘modular’ patterns, or in an extreme case, ‘segregated’ patterns. In these scenarios, specific bacterial taxa would be associated with distinct groups of meerkats or even exclusively with individual meerkats, suggesting the presence of separate subgroups of microbial communities rather than a continuous gradient (Strona & Veech, 2015).

Using the R package *Bipartite* (Dormann et al., 2014), we constructed ‘*m* × *n*’‐ networks, connecting one set of nodes representing bacterial ASVs (*m*) to another set of nodes containing individual meerkat IDs (Figure S1). These were constructed for the entire dataset, including all 146 meerkats and the 119 ASVs, and separately for meerkats within each of the three study periods, including 108 ASVs in period I, 95 in period II and 102 in period III (Table S1). We thus analysed four bipartite networks. For these, edges connecting ASVs to meerkats were included as values in the ‘*m* × *n*’ network matrix. These were either weighted by the relative abundance of ASVs within meerkats (for those meerkats on which we had multiple samples, we used average values across all samples) or unweighted in the sense that they were effectively treated as if a given ASV (*m*) was present (1) or absent (0) among meerkats (*n*). All statistical tests were two‐tailed, and we set *p*‐values <0.05.

For each network, we calculated degrees of nestedness using the R package *vegan* (Oksanen et al., 2013). Specifically, we used the function ‘nestedNODF’ to calculate both weighted (WNODF) and unweighted (NODF) indices of nestedness of bacterial ASVs among meerkats. We also used the function ‘nestedtemp’ to calculate the bipartite matrix temperature (Temp) as an indicator of the degree of deviation from entropy or randomness in the distribution of links between ASVs (rows) and meerkats (columns). For unweighted measures of nestedness and matrix temperature, we used the ‘oecosimu’ function to perform permutation‐based significance tests comparing observed values to a distribution of nestedness values generated after 1000 simulations of randomly swapping matrix (network) rows and columns. To determine whether bacterial communities exhibited modular or segregated structures, we used the ‘computeModules’ function in the bipartite package in R. This estimates a weighted measure of Newman's modularity, ranging from 0 (non‐modular network) to 1 (perfectly modular or segregated network). We then used prenetwork randomisation tests, or an edge swapping algorithm, to determine whether the observed modularity scores were significantly greater than the scores of 1000 simulated networks generated by randomly swapping the edges of the network (Farine & Carter, 2022).

#### 2.6.2 Does group membership and/or kinship influence bacterial community composition?

To determine the relative importance of group membership and kinship in shaping bacterial communities, we first calculated the degree of *β* diversity (dis)similarity in bacterial ASV prevalence and relative abundance between each pair of meerkats. We did this using the function beta.pair available in the *betapart* package in R (Baselga & Orme, 2012). From the overall ‘*m* × *n*’ bipartite matrix, this function was used to generate a unipartite ‘*n* × *n*’‐ matrix in which the rows and columns were individual meerkats and the cells indicated Jaccard's indices of pairwise bacterial dissimilarity. We then performed multivariate matrix regression using a quadratic assignment procedure (MR‐QAP; Dekker et al., 2007; Farine & Carter, 2022), using the *sna* R package (Butts, 2008). The outcome matrix was the *n* × *n β* diversity dissimilarity matrix. As predictor matrices, we included information on the animals' group membership (i.e. whether pairs of meerkats belonged to the same or different groups), their relatedness as indicated by their pairwise relatedness coefficients, and a ‘study period’ matrix indicating whether pairs of meerkats were sampled during the same time period, to control for its potential effect on bacterial (dis)similarity. We also ran eight univariate MR‐QAPs, one for each of the eight study groups, to assess within‐group evidence for the effects of kinship alone (predictor matrices) on bacterial *β* diversity similarity (outcome matrices).

#### 2.6.3 What is the relative importance of social organisation compared to biological characteristics and environmental factors in influencing microbiomes?

We ran JSDMs using the R package *Hmsc* (Tikhonov et al., 2020) to determine the relative importance of different host social factors, biological characteristics and environmental factors on gut bacterial communities. In each JSDM, rows were sample IDs and columns were bacterial ASVs (Table S1). As sample‐specific covariates, we included all co‐variates summarised in Table 1 except kinship. We did not include kinship because it is a dyadic measure and, therefore, was more appropriately assessed using the network‐based MR‐QAP tests described above rather than using the JSDMs. In the latter, we could only evaluate individual‐level covariates. Additionally, we later concluded that the exclusion of kinship from the JSDMs would not affect our findings, as the MR‐QAP tests showed that kinship had a significantly lower overall effect on bacterial similarity compared to group membership (see Section 3).

We ran four JSDMs in total. First, we ran a ‘full model’ where we included each of the three social factors, four biological covariates and four environmental covariates. We also ran three ‘covariate‐specific models’, one each including only social (without kinship), biological and environmental covariates. To control for repeated sampling of the same individuals and for variation between study periods, we also included animal ID and period ID (three categories: 2006–2007, 2014–2015, 2016–2017) as random effects.

All four models were fitted with the Bayesian inference criterion using the ‘normal’ distribution function. For each model, we used the default priors and ran two chains of 1500 iterations each, from which we removed the first 500 iterations as burn‐in and thinned the remainder to give 1000 posterior samples per chain per model. We used visual inspection of the MCMC plots and calculations of the Gelman‐Rubin diagnostics to assess model convergence for each model. We then implemented a corrected least squares regression criterion (or CSR^2^ score) to select and further interpret the best fit of the four models. From this ‘best‐fit’ JSDM, we assessed the relative contributions of the covariates using two approaches. In the first approach, we used generalised linear mixed models (GLMMs) with a beta distribution function to compare the mean absolute values of effect sizes across covariates. In this analysis, the outcome variables were the effect sizes of the covariates on the relative abundance of each bacterial ASV, as derived from the JSDM outputs. As predictors, we included covariate type as a main effect, with bacterial ASV and covariate category as random effects. Specifically, we ran three generalised linear mixed models (GLMMs) to compare the mean effect sizes of the three social covariates against the effect sizes of the four biological and four environmental covariates. We also ran three additional beta‐distributed GLMMs to compare the mean effect sizes of covariates within the same category (i.e. social, biological or environmental covariates separately). As a second approach, we extracted the relative variance contributions of the covariates from the best‐fit JSDM and compared their influence on the observed distribution of relative bacterial ASV abundance.

#### 2.6.4 Did bacterial ASVs show strong propensities to co‐occur within hosts?

To determine the influence of bacterial phylogenetic relationships on communities, we also included ASV‐ASV phylogenetic distances (branch lengths) in all JSDMs to quantify phylogenetic signal, that is, the extent to which more closely related bacterial ASVs tend to co‐occur in the same meerkats compared to more distantly related ASVs. Through the JSDMs, we also assessed whether and which bacterial ASVs formed strongly positive or negative associations that were indicative of their overall propensities to co‐occur within individual meerkats. For this, we extracted and interpreted a ‘residual’ co‐occurrence matrix of positive and/or negative ASV‐ASV associations from the best‐fit JSDM. We also used this matrix to assess the types of relationships between a subset of bacterial ASVs that were also the ones most strongly influenced by meerkats' social organisation.

## 3 RESULTS

### 3.1 Bacterial communities showed a predominantly nested, weakly modular structure

We observed non‐random patterns of bacterial ASV co‐occurrence in the gut microbiota of wild meerkats (Figure S1). Across all three study periods, as well as within each individual period, bipartite co‐occurrence networks of bacterial ASVs associated with individual meerkats exhibited strongly nested structures (Figure S2A–D). This is indicated both by the significantly high nestedness temperature (Temp_overall dataset_ = 28.98, *p* < 0.001; see Figure S2B–D for values from period‐specific networks) and NODF (NODF_overall_ = 83.89, *p* < 0.001; Figure S2B–D for values from period‐specific networks) values from unweighted networks and the high NODF values from the weighted networks (w.NODF_overall dataset_ = 48.55; see Figure S2B–D for values from period‐specific networks). In other words, many ASVs were present in most or all meerkats, while others were found only in a minority of individuals. Correspondingly, the networks also showed lower modularity, suggesting that only a small number of bacterial ASVs clustered uniquely by group or study period (Figure S2A–D).

### 3.2 Group membership had a stronger effect than kinship on bacterial community similarity

We found that animal group membership had a strong effect on bacterial communities. The MR‐QAP test showed that meerkats from the same group had more similar bacterial compositions, that is, a higher Jaccard pairwise *β* diversity, than those from different groups (*β* = 0.26, *p* < 0.001; Table S3), independent of the effect of study period. The latter also had a significant effect on bacterial similarity, with communities also unsurprisingly being more similar within the same study period than across different periods (*β* = 0.26, *p* < 0.001; Table S3). In contrast, relative to group membership and study period, kinship had no effect on bacterial composition across groups (Table S3). However, our univariate MR‐QAPs *within* each group revealed that kinship strongly influenced bacterial similarity in at least five of the eight groups. Specifically, the degree of relatedness between pairs of meerkats was positively associated with bacterial community similarity in three groups (Group I: *β* = 0.26, *p* < 0.01; Group II: *β* = 0.13, *p* = 0.04; Group V: *β* = 0.59, *p* < 0.01), with two additional groups showing nonsignificant trends (Groups III and VI) (Table S4).

### 3.3 Social, biological and environmental factors influenced bacterial abundance, with group membership, age and sampling time having the strongest effects

**FIGURE 1:**
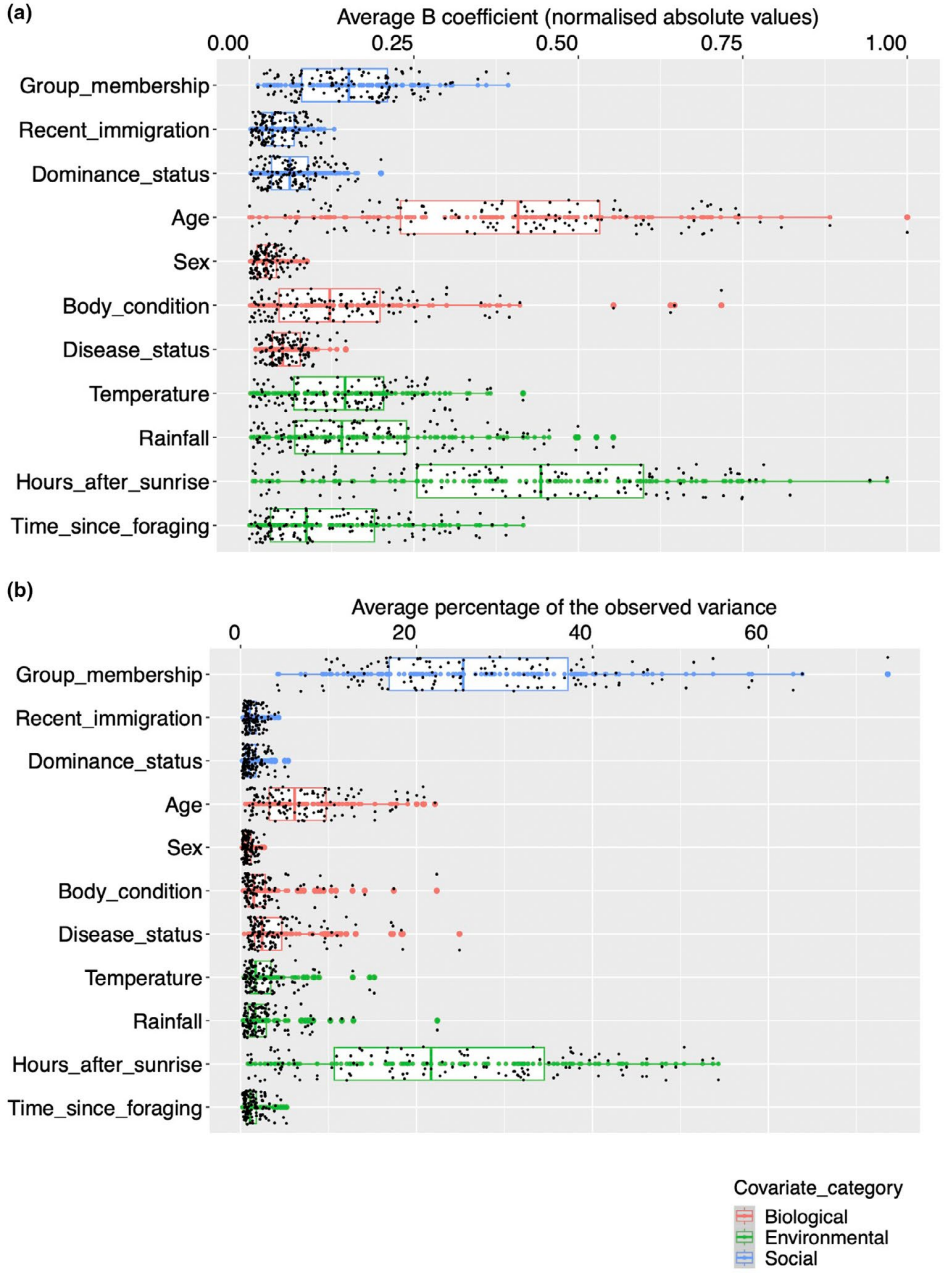
Boxplots showing (a) effect sizes (standardised absolute *β*‐coefficients) and (b) variance contributions (percentage values) of the effects of social, biological (demographic, health‐related) and environmental (climatic, temporal) factors on the relative abundance of 119 bacterial amplicon sequence variants in meerkats.

JSDMs revealed that the social, biological and environmental covariates influenced bacterial abundance. Using the CSR^2^ model selection criterion, the full model including all covariates was a significantly better fit than subset models including only certain categories of covariates (Figure S3; Table S5). GLMMs comparing the effect sizes of covariates within the same categories revealed important differences. Among social covariates, group membership had the largest effect, followed by dominance status, with recent immigration into the group having the smallest effect. Among host biological variables, age had the largest mean effect size, followed by body condition and disease status, with sex having the smallest effect. Among environmental variables, sampling time after sunrise had the largest effect, followed by rainfall and temperature, and finally hours since foraging (Figure 1a; Table S6A–C).

The GLMMs comparing the effect sizes of each social covariate with each biological and each environmental variable showed that group membership had a significantly smaller effect than host age and sampling hours after sunrise, which had the largest mean effect sizes on bacterial abundance of all 11 covariates (Figure 1a; Table S6D). However, group membership had a significantly larger effect than the other biological covariates (sex, body condition and disease status) and the temporal covariate, time elapsed since foraging. However, group membership was not significantly different from the effect sizes of the two climate‐related covariates, temperature and rainfall (Figure 1a; Table S6D). In contrast to group membership, recent immigration and dominance status had significantly smaller effect sizes compared to all four environmental covariates and some biological variables, namely age and body condition, and partly disease status (Figure 1a; Table S6E,F). However, these two social covariates had significantly larger effects than sex, which had the smallest effect size on bacterial abundance among the 11 covariates (Figure 1a; Table S6E,F).

**FIGURE 2:**
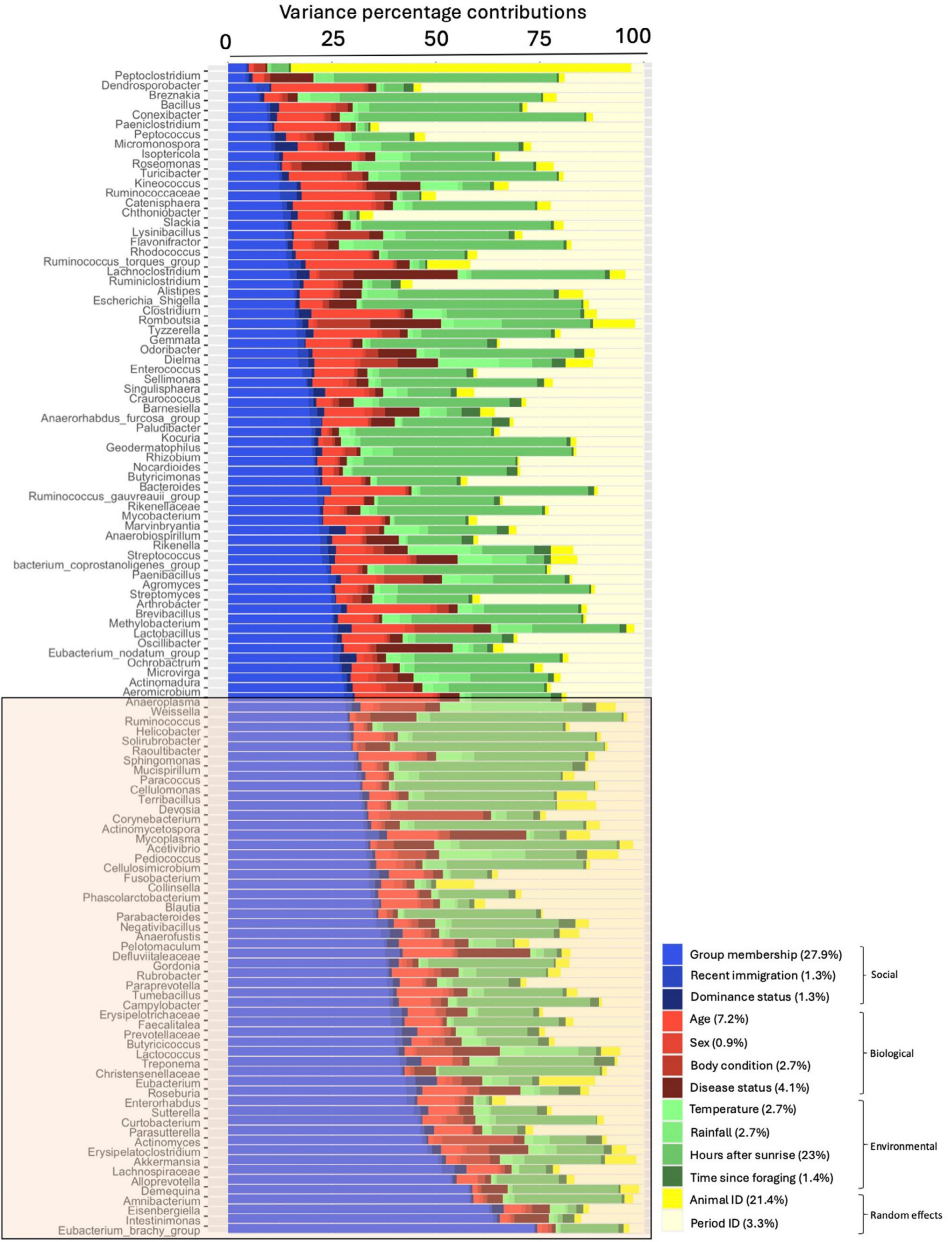
Plot of proportions of variance explained by the joint‐species distribution model, depicting the relative contributions of social, biological and environmental covariates to the relative abundance of bacterial amplicon sequence variants (ASVs). Values in brackets indicate the mean percentage contribution of each covariate. ASVs are ordered in ascending order of the percentage contribution of meerkat group membership to their relative abundance. The rectangular frame indicates bacterial ASVs on which group membership had a particularly strong effect, that is, greater than its mean contribution of 27.9%.

Comparisons of variance proportion values from the JSDM showed that, on average, group membership (27.9%) and sampling time after sunrise (23%) accounted for the largest proportions of variance in ASV abundance (Figures 1b and 2). Meerkat animal ID, included as a random effects term in the model, was the third highest contributor (21.4%). In contrast, other factors had much smaller effects, indicating more stable contributions that were also, on average, markedly lower in magnitude than those of group membership and sampling time after sunrise. Among these factors, meerkat age was the fourth largest contributor to the variance in ASV abundance (7%), despite having a high mean effect size. This was followed by Period ID (3.3%), which, like Animal ID, was included as a random effect. Social covariates, such as recent immigration (1.3%) and dominance status (1.3%), had minimal impact, with sex being the smallest contributor at 0.9% (Figures 1b and 2).

Figure 2 shows the variance contributions of each covariate towards each bacterial ASV. This includes 55 bacterial ASVs for which group membership showed a particularly strong variance contribution that was greater than its average contribution of 27.9% (range: 27.94%–73.57%; see also Table S2).

### 3.4 Bacterial communities showed weak phylogenetic signals, but showed strong ASV‐ASV co‐occurrence independent of phylogeny and host covariates

**FIGURE 3:**
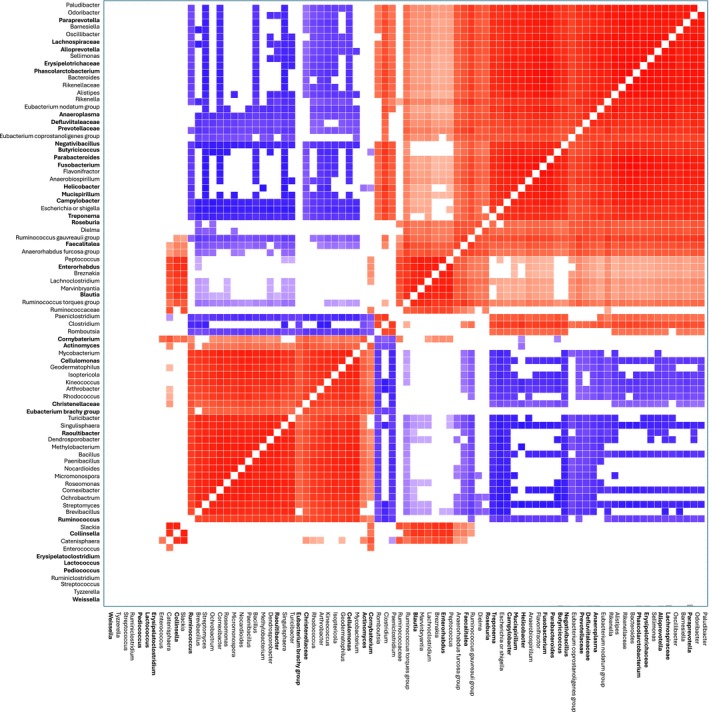
Heat map of ‘residual’ correlations between bacterial amplicon sequence variants (ASVs) from the joint‐species distribution model. Rows and columns indicate the most abundant 83 ASVs (>10% in all study periods, Table S2). Coloured cells indicate both positive (red) and negative (blue) bacterial residual correlations that reached significance (≥95% confidence). Taxa in bold are 32 ASVs on which group membership had a particularly strong effect that was greater than its mean variance contribution of 27.9% (see Figure 2).

The best‐fit JSDM showed no evidence of phylogenetic signals, that is, for more closely related ASVs to co‐occur within the same host (*ρ* = 0.25, *p* = 0.21). The model revealed both significantly positive and significantly negative ‘residual’ correlations between many pairs of bacterial ASVs that persisted after accounting for the effects of phylogeny and of the social, host biological and environmental covariates. Figure 3 shows a heat map of this residual co‐occurrence matrix, constructed for just the 83 ‘most prevalent’ bacterial ASVs (Table S2). Among these, we identified 32 ASVs that were among the most strongly influenced by group membership (Figure 2). Examination of these ASVs (Figure 3) revealed that most bacterial taxa showed significantly positive associations with each other, including beneficial bacteria like *Mucispirillum*, *Alloprevotella*, *Roseburia* and *Blautia*, which co‐occurred with potentially harmful genera such as *Fusobacterium* and *Campylobacter*. In contrast, *Cellulomonas*, *Christensenellaceae* and *Ruminococcus* showed negative associations with ASVs like *Alloprevotella*, *Roseburia*, *Negativibacillus* and *Butyricicoccus*. A few taxa, such as *Weissella*, *Lactococcus*, *Pediococcus* and *Erysipelatoclostridium*, showed no significant associations with any other taxa.

## 4 DISCUSSION

Our study addresses key gaps in understanding the ecology of wildlife microbiomes by empirically testing aspects of the social microbiome concept (Baniel & Charpentier, 2024; Sarkar et al., 2020). Among wild meerkats in the Kalahari, we found that microbiomes were strongly influenced by social group membership, more so than kinship. Group membership also shaped the co‐occurrence of many beneficial bacteria, as well as a few ASVs that may potentially harm or detrimentally affect host health. Additionally, meerkats' age and sampling time after sunrise had significant effects on bacterial abundance. In contrast, individual‐level aspects of social organisation, such as sex, health‐related variables and exposure to environmental factors like climate had weaker effects on bacterial abundance and co‐occurrence. Below, we discuss our findings in the context of the social microbiome from an evolutionary ecological perspective and explore their implications for meerkat health.

Bacterial communities exhibited nested structures: Many ASVs were present in nearly all individuals sampled, while others appeared in fewer. This suggests that, while many meerkats harboured high microbial diversity, some had lower diversity consisting of ASVs that were subsets of the overall community. This pattern aligns with another finding: The networks showed low to moderate modularity based on meerkat group membership or study period. Specifically, a few bacterial taxa clustered within distinct groups of hosts, likely meerkats within the same (as opposed to different) groups. This indicates that meerkats within the same group may harbour unique bacterial communities or subcommunities that are more likely to be shared with others in that group. The observation of a globally nested bacterial community structure with localised modularity provides crucial empirical support for the concept of ‘core’ microbiota—microbes that are found across the majority of hosts in a population—versus ‘non‐core’ microbiota, which may vary more dynamically in response to transient environmental factors (Grieneisen et al., 2017; Risely, 2020). Furthermore, the clear architecture of meerkat microbiomes strengthens the premise that bacterial abundance and co‐occurrence are influenced by deterministic factors, rather than being driven solely by stochastic processes (Hayashi et al., 2024; Zhou & Ning, 2017).

Among the aspects of social organisation that we examined, group membership had the strongest effect on the similarity of meerkats' bacterial profiles, more so than kinship (MR‐QAP analyses) and individual‐level social factors like recent immigration and dominance status (JSDM analyses). Meerkats within the same group shared more similar ASV types and relative abundances compared to meerkats from different groups. These similar bacterial ‘profiles’ between group members may result from horizontal bacterial sharing or transmission through social interactions (either contact or non‐contact), or from the independent acquisition of the same bacteria due to shared exposure to environmental factors (Debray et al., 2024; Sarkar et al., 2020). The finding that meerkat group membership had a stronger effect on their microbiomes than their degree of relatedness or kinship suggests that meerkat microbiomes may be more strongly influenced by horizontal social or shared spatial transmission, rather than by vertical transmission between closely related mother–offspring pairs during parturition and/or milk ingestion (reviewed in Sarkar et al., 2020). However, the impact of relatedness‐mediated vertical transmission in meerkats cannot be completely ruled out, as microbiomes seemed to be more similar among closer kin within at least five of the eight groups. It has been speculated that kinship might play a greater role in shaping the social microbiome in taxa with higher overall genetic relatedness within groups (e.g. eusocial insects, cooperatively breeding meerkats and naked mole rats: reviewed in Sarkar et al., 2020). Conversely, species with relatively lower within‐group genetic relatedness might be expected to show a greater influence of social contact patterns independent of genetic relatedness on microbiome similarity, as shown in primates like red colobus monkeys (*Cercopithecus badius*, Goodfellow et al., 2019), chimpanzees (Degnan et al., 2012) and more recently humans (Beghini et al., 2024). Similar between‐group variation may also occur within a species, as appears to be the case here. In meerkats, the observed variation in kinship effects on microbiomes may reflect differences in group size or female‐to‐male sex ratios, which in turn influence genetic relatedness within groups. Testing this would require microbiome data from a larger number of groups. As such, our finding that group membership has a stronger influence on bacterial similarity than kinship in a cooperatively breeding species challenges this argument. Indeed, high genetic similarity among meerkats—especially within the same group compared to between groups—could facilitate more similar bacterial acquisition or ‘colonisation’ among genetically similar hosts. This might occur because genetically similar animals may also experience more similar socioecological pressures, respond to these pressures in similar ways and/or have a higher likelihood of vertical transmission from mother to offspring (Sarkar et al., 2020, 2024). To empirically test the ‘microbial colonisation’ hypothesis, it is essential to conduct comparative studies within and between species to examine the relationship between genetic relatedness and microbiome similarity among individuals within groups and populations. It is also possible that the effects of host genetic relatedness might be more apparent if we examined the impact of meerkat genealogies on strain‐level (rather than genus‐level) microbial metagenomic composition. Both of these approaches were beyond the scope of this study but offer important future directions for better understanding the role of genetic relatedness in shaping animal microbiome communities.

The JSDM approach further confirmed the strong impact of meerkat group membership on bacterial communities, specifically on the acquisition of several bacterial ASVs with known health benefits to animals, but also a few potentially detrimental ASVs (Fackelmann et al., 2023; Risely et al., 2023). For example, *Weissella* is linked to probiotic and anti‐inflammatory properties (Ahmed et al., 2022), *Mucispirillum* protects the gut and inhibits *Salmonella* infection (Herp et al., 2019), and *Blautia* plays a key role in alleviating anti‐inflammatory and metabolic diseases (Liu et al., 2021). *Roseburia* is a well‐known beneficial bacterium in the human gut (Nie et al., 2021). It produces short‐chain fatty acids (SCFAs), especially butyrate, which are crucial for maintaining gut health. However, potential harmful bacteria were also linked to group membership. For instance, *Fusobacterium* has been associated with tuberculosis (Tb) infection and poor body condition in meerkats and is often highly abundant in diseased individuals suffering from a dysbiotic gut microbial community in wildlife (Risely et al., 2022, 2023). *Campylobacter* is associated with reproductive disorders, gastric ulcers and diarrheal disease (Riddle et al., 2012). A subsequent examination of these ASVs revealed that the majority of these bacterial taxa exhibited significantly positive associations with each other. These included beneficial bacteria such as *Mucispirillum*, *Alloprevotella*, *Roseburia* and *Blautia*, which appeared to positively co‐occur with each other but also with several genera including taxa potentially detrimental to host health, such as *Fusobacterium* and *Campylobacter*, which were all also positively associated with each other. In summary, these findings suggest that meerkat socioecology is associated with the sharing of predominantly beneficial bacteria, as well as a few ASVs that may be harmful to host health. This provides strong empirical support for the idea that the sharing of beneficial bacteria has been a positive evolutionary driver of animal sociality (Archie & Tung, 2015; Lombardo, 2008; Nunn et al., 2015), and more broadly, supports the ‘social determinants of health’ framework (Snyder‐Mackler et al., 2020).

Along with social factors like group membership, biological and environmental factors also impacted bacterial abundance. Meerkat age had the strongest effect on mean bacterial abundance, while animal ID influenced variance and sampling time after sunrise affected both mean and variance. Climatic variables such as rainfall and temperature and body condition had comparable effect sizes to group membership.

Bacterial communities were strongly influenced by the time of sampling after sunrise, meerkat age and the random effect of individual animal ID. These findings are consistent with previous research on wildlife microbiomes, which highlight strong associations between temporal changes and microbial turnover. Notably, the pronounced effect of sampling time on bacterial abundance supports earlier evidence of microbiome oscillations linked to circadian rhythms (Risely et al., 2021). Similar temporal patterns have been observed in other taxa, including fish, chickens, mice and humans, as reviewed by Schmid et al. (2023). Moreover, the strong effect of animal age on bacterial abundance not only supports our previous findings in meerkats (Risely et al., 2022) but also contributes to a growing body of evidence from other group‐living mammals, including yellow‐bellied marmots (Pfau et al., 2023), spotted hyenas (Rojas et al., 2023), yellow baboons (Dasari et al., 2025), chimpanzees (Reese et al., 2021) and humans (O'Toole & Jeffery, 2015). Notably, while meerkat age affected mean bacterial abundance, it had less impact on variance. This pattern aligns with our earlier observation that diurnal oscillations in relative bacterial abundance were generally less pronounced in older meerkats—whose microbiomes appeared more stable—compared to younger individuals, which exhibited greater fluctuations (Risely et al., 2021, 2022).

Compared to meerkat group membership, age and sampling time after sunrise, animals' health (e.g. body condition, *Tb* infection status) and other environmental factors (e.g. temperature, rainfall, period ID, time elapsed after foraging) had relatively weaker effects on bacterial abundance. One possible explanation is that these factors may influence the abundance of specific bacterial taxa rather than overall bacterial composition. For instance, *Tb* exposure and/or infection stages have recently been associated with shifts in particular taxa, including declines in beneficial *Lactococcus* spp. and *Pediococcus* spp., and the co‐occurrence of potentially harmful *Bacteroides* spp. (Risely et al., 2023). Another possibility is that the detectability of some of these effects depends on the spatio‐temporal scale of sample collection. For example, climate‐related variables and seasonal variation may require consistent long‐term sampling to detect patterns (as shown in our previous research; Risely et al., 2023), whereas short‐term sampling may be more appropriate for capturing the influence of prey diversity and abundance on the gut microbiome—an area yet to be explored in this population (Jubber et al., 2025).

Individual‐level aspects of meerkat social organisation, specifically recent immigration into groups and their dominance status, as well as the individual sex, had the weakest effects on relative bacterial abundance. Notably, there is substantial intraspecific variation in patterns of agonistic and affiliative behaviour among meerkats. Dominant breeding pairs, for instance, typically engage more frequently in both types of social interactions and are more socially integrated into their groups than subordinates (Clutton‐Brock & Manser, 2016; Madden et al., 2009, 2011). Therefore, differences in the nature and frequency of social interactions—particularly in direct and indirect social network connectedness—may offer a better explanation for microbiome variation than broad classifications such as dominant versus subordinate, resident versus immigrant or male versus female. Moreover, the relatively weak effect of recent immigration, compared to group membership, suggests that immigrant individuals' microbiomes may rapidly adjust in response to the socio‐ecological changes following immigration. Confirming this would require more frequent temporal sampling, including longitudinal comparisons of microbial profiles between immigrants and long‐term residents across key phases: immigration, social integration and tenure within the new group (Grieneisen et al., 2017).

In summary, our study highlights the multifaceted nature of microbial diversity and supports the idea that the social microbiome is shaped by multiple deterministic factors. This broader perspective, supported by our study, makes the social microbiome concept more comprehensive and realistic, particularly in wild populations where environmental interactions, disease exposure and other stressors likely shape microbial composition. Our study advances previous research on the ecology of social microbiomes, which has largely focused on detecting socially or network‐mediated microbial sharing and transmission (Debray et al., 2024; Sarkar et al., 2020). Rather than concentrating solely on predispositions to social transmission, we assessed and empirically confirmed the relative contributions of multiple deterministic factors in shaping animal microbiomes—including social organisation, individual biological traits, exposure to abiotic environmental conditions and ecological and evolutionary microbe–microbe associations driving co‐occurrence patterns. We demonstrated the utility of JSDMs as powerful quantitative tools for analysing microbial community structure and composition across social and macro‐ecological scales. This approach has also been applied to understand pathogen co‐occurrence in African lions (Fountain‐Jones et al., 2019) and viral communities in wild rhesus macaques (Balasubramaniam et al., 2025). While our findings highlight the strength of JSDMs in uncovering broad ecological patterns, several limitations—such as the absence of detailed social interaction or shared space‐use data between individuals, restricted temporal resolution of microbial sampling and reliance on 16S rRNA for genus‐level ASV identification—limit finer scale interpretations of microbial transmission dynamics and function. Additionally, current JSDMs are not equipped to model sequential ASV acquisition or indirect effects among covariates. Addressing these limitations in future work, for example by integrating structural equation models or metagenomic approaches, will be essential to further disentangle the complex interactions shaping host‐associated microbial communities. Such integrative assessments of Kalahari meerkats—and other vulnerable wildlife systems—are also critical from a conservation perspective, especially in light of the increasing anthropogenic pressures that shape social microbiomes and influence associated health outcomes in wild populations.

## AUTHOR CONTRIBUTIONS

Krishna Balasubramaniam designed the study, performed data analysis and wrote the manuscript, with Nadine Mueller‐Klein and Simone Sommer providing consistent input through the analytical and writing phases. Nadine Mueller‐Klein, through facilities and resources of Simone Sommer, supervised the processing of all faecal samples for the characterisation and evolutionary (phylogenetic) analysis of microbiomes. Timothy H. Clutton‐Brock and Marta B. Manser initiated and supervised the long‐term meerkat project and data collection, with Tim Vink being particularly involved in the collation and processing of meerkat social and biological datasets used in the study. Simone Sommer supervised the entire study, acquired funding for microbiome investigation and performed project administration.

## CONFLICT OF INTEREST STATEMENT

The authors declare no conflicts of interest.

## Supporting information


**Figure S1.** Possible, non‐mutually exclusive patterns of bipartite network associations between bacterial amplicon sequence variants (ASVs) and meerkats (adapted from Strona & Veech, 2015).

**Figure S2.** Temperature plots showing the ‘incidence’ of ASV in meerkats.

**Figure S3.** Comparisons of model‐fit parameters (mean CSR^2^ values from bacterial ASVs) from across four JSDMs, that is, across a full model that included all covariates, and the three covariate‐specific models.

**Table S1.** Meerkat groups investigated during three different study periods.

**Table S2.** Final list of 119 bacterial ASVs (phylum, family & genus) analysed in the study.

**Table S3.** Multivariate MR‐QAP to examine the effects of group membership (‘same’ vs. ‘different’ groups) and kinship (Wang's relatedness coefficient) on the degree of Jaccard pairwise *β*‐diversity similarity in bacterial community composition (ASV abundance and diversity). Period of study was included as a control variable.

**Table S4.** Univariate MR‐QAPs to examine within‐group effects of kinship (Wang's relatedness coefficient) on the degree of Jaccard pairwise *β*‐diversity similarity in bacterial community composition (ASV abundance and diversity).

**Table S5.** Parameter estimates from a generalised linear mixed model (GLMM) used to evaluate the fit of CSR^2^ values (outcome variables) of four joint‐species distribution models (JSDMs).

**Table S6.** Parameter estimates from GLMMs used to compare the mean effect sizes of covariates, on the relative abundance of bacterial ASVs.

## Data Availability

The sequences used in the study for bacterial phylogenetic reconstruction are all available at NCBI BioProject PRJNA764180. All data used for this project and R code to replicate our analyses have been made available publicly via figshare: https://doi.org/10.6084/m9.figshare.28496426.
