# Supplementary material for: Impact of animal socioecology on gut microbial communities: Insights from wild meerkats in the Kalahari
Source: J Anim Ecol. 2025 Oct 30;94(12):2687–703. doi: 10.1111/1365-2656.70168 (PMC12673242; doi:10.1111/1365-2656.70168)
Supplement: Supplementary file 1 — Figure S1. Possible, non‐mutually exclusive patterns of bipartite network associations between bacterial amplicon sequence variants (ASVs) and meerkats (adapted from Strona & Veech, 2015). [file JANE-94-2687-s007.docx]

**Supporting Figure 1:** Possible, non-mutually exclusive patterns of bipartite network associations between bacterial Amplicon Sequence Variants (ASVs) and meerkats (adapted from Strona & Veech, 2015). A ‘nested’ structure (A) would indicate a gradient of bacterial species richness, i.e. widespread abundance of many bacterial ASVs across many meerkats with just a few ASVs that occur in subsets of meerkats. On the other hand, (B) a ‘modular network’, or in an extreme case a (C) ‘segregated network’ would indicate that specific bacterial ASVs would be associated with distinct groups of meerkats (B), or even exclusively with individual meerkats (C), suggesting the presence of separate subgroups of bacterial communities.
