# Supplementary material for: Impact of animal socioecology on gut microbial communities: Insights from wild meerkats in the Kalahari
Source: J Anim Ecol. 2025 Oct 30;94(12):2687–703. doi: 10.1111/1365-2656.70168 (PMC12673242; doi:10.1111/1365-2656.70168)
Supplement: Supplementary file 2 — Figure S2. Temperature plots showing the ‘incidence’ of ASV in meerkats. [file JANE-94-2687-s001.docx]

**Supporting Figure 2:** Temperature plots showing the 'incidence' of ASV in meerkats. Plots were constructed using (A) the full dataset of 119 ASVs and (B - D) separately for animals within each of the three study periods with 108, 95 and 102 ASVs respectively. Cells in red represent higher ASV incidence (in columns). Each plot is accompanied by corresponding indices of nestedness (both weighted and unweighted nested NODF and unweighted matrix temperature or 'Temp') and modularity of the ASV-meerkat bipartite co-occurrence networks. P-values were estimated from comparisons of the observed index with a distribution of indices generated from 1000 pre-network simulations.
