# Supplementary material for: Impact of animal socioecology on gut microbial communities: Insights from wild meerkats in the Kalahari
Source: J Anim Ecol. 2025 Oct 30;94(12):2687–703. doi: 10.1111/1365-2656.70168 (PMC12673242; doi:10.1111/1365-2656.70168)
Supplement: Supplementary file 3 — Figure S3. Comparisons of model‐fit parameters (mean CSR2 values from bacterial ASVs) from across four JSDMs, that is, across a full model that included all covariates, and the three covariate‐specific models. [file JANE-94-2687-s002.docx]

**Supporting Figure 3:** Comparisons of model-fit parameters (mean CSR^2^ values from bacterial ASVs) from across four JSDMs, i.e. across a full model that included all covariates, and the three covariate-specific models.


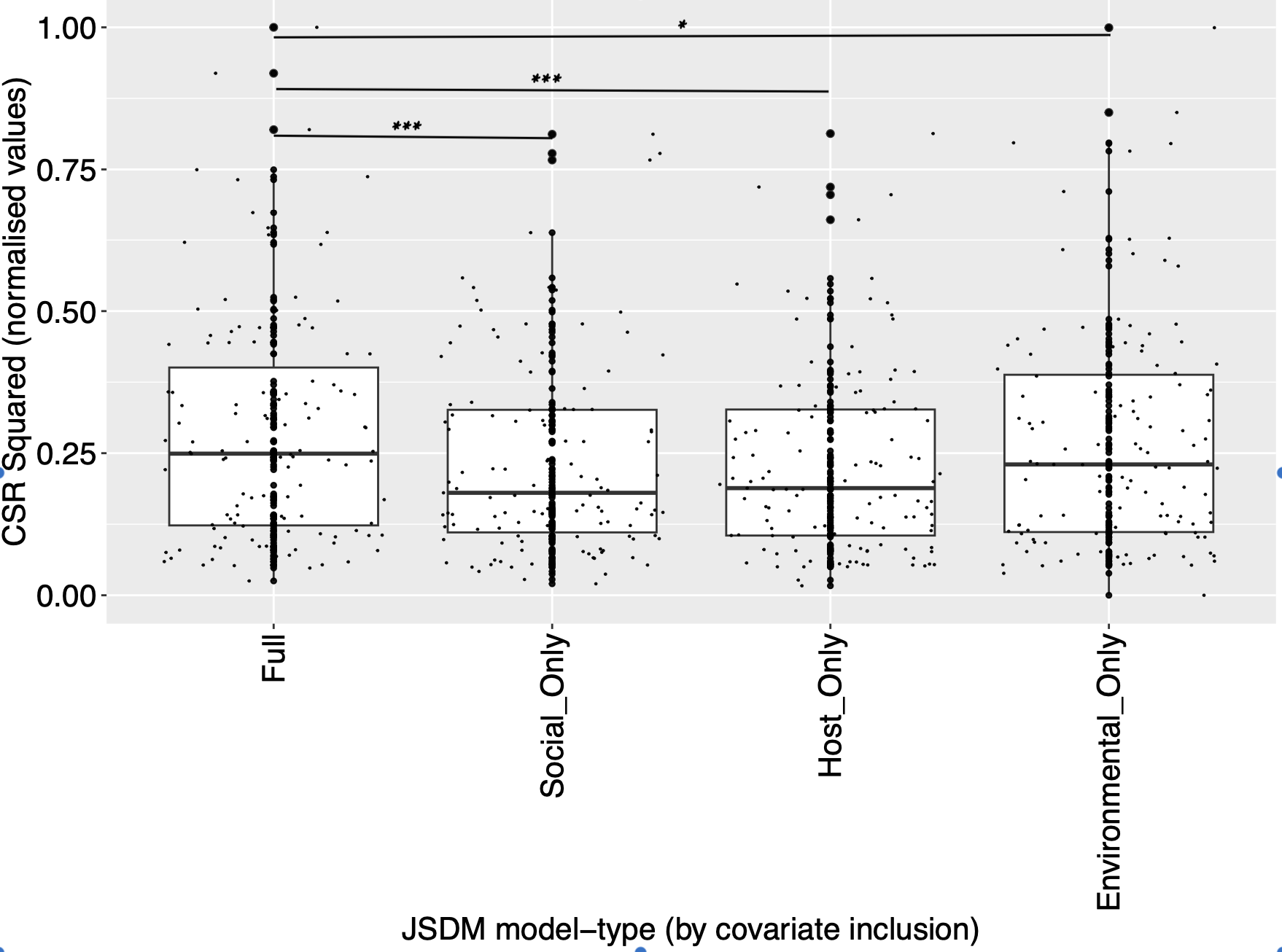


***p < 0.001; *p < 0.05
