# Supplementary material for: Impact of animal socioecology on gut microbial communities: Insights from wild meerkats in the Kalahari
Source: J Anim Ecol. 2025 Oct 30;94(12):2687–703. doi: 10.1111/1365-2656.70168 (PMC12673242; doi:10.1111/1365-2656.70168)
Supplement: Supplementary file 4 — Table S1. Meerkat groups investigated during three different study periods. [file JANE-94-2687-s008.docx]

**Supporting Table 1:** Meerkat groups investigated during three different study periods. The number of samples and individuals sampled per meerkat group and study period are indicated.

| **Study period** | **Meerkat group** | **Average group size (& range) across the study period** | **Number of samples** | **Number of unique meerkats (males, females) sampled** |
| --- | --- | --- | --- | --- |
| **Period I: 2006-2007** | I | 36 (31 – 41) | 79 | 20 (13, 7) |
|  | II | 36 (32 – 40) | 97 | 20 (11, 9) |
|  | III | 55.5 (54 – 57) | 79 | 23 (14, 9) |
|  | IV | 21 (20 – 22) | 51 | 11 (5, 6) |
| **Period II: 2014-2015** | VII | 29 (28 – 30) | 40 | 10 (3, 7) |
|  | VIII | 28 (27 – 29) | 51 | 12 (7, 5) |
| **Period III: 2016-2017** | V | 39 (36 – 42) | 62 | 24 (12, 12) |
|  | VI | 30.5 (29 – 32) | 69 | 26 (18, 8) |
| **Total** | **8** |  | **528** | **146** |
