# Supplementary material for: Impact of animal socioecology on gut microbial communities: Insights from wild meerkats in the Kalahari
Source: J Anim Ecol. 2025 Oct 30;94(12):2687–703. doi: 10.1111/1365-2656.70168 (PMC12673242; doi:10.1111/1365-2656.70168)
Supplement: Supplementary file 5 — Table S2. Final list of 119 bacterial ASVs (phylum, family & genus) analysed in the study. [file JANE-94-2687-s005.docx]

**Supporting Table 2: Final list of 119 bacterial ASVs (phylum, family & genus) analysed in the study.** ASVs with a prevalence ≥ 10% across the overall dataset were selected for inclusion in this table (n= 119). Among those, 108 unique ASVs met the ≥ 10% prevalence criterion in Period I, 95 in Period II, and 102 in Period III, and were included in the period-specific network analyses (marked with ‘1’ in the corresponding columns). For constructing the residual co-occurrence network using the coefficients from the JSDM, 83 ASVs were selected based on the highest prevalence (≥ 10% in all study periods, indicated by ‘x’ in the relevant column). ASVs highlighted in grey (n= 55) were most strongly influenced by meerkat social group membership, where group membership contributed a higher-than-average proportion of the overall variance (see Figure 3 in the main manuscript).

|  | **119 Bacterial ASVs** | | | **ASVs ≥ 10% prevalence in all Periods**  **(n= 83)** | | **ASVs ≥ 10% prevalence in Period I (n= 108)** | **ASVs ≥ 10% prevalence in Period II**  **(n= 95)** | **ASVs ≥ 10% prevalence in Period III**  **(n= 102)** |
| --- | --- | --- | --- | --- | --- | --- | --- | --- |
| **Phylum** | **Family** | | **Genus** | |  |  |  |  |
| Firmicutes | | Ruminococcaceae | *Acetivibrio* | |  | 1 | 0 | 1 |
| Actinobacteria | | Thermomonosporaceae | *Actinomadura* | |  | 1 | 1 | 0 |
| Actinobacteria | Actinomycetaceae | | *Actinomyces* | | x | 1 | 1 | 1 |
| Actinobacteria | Pseudonocardiaceae | | *Actinomycetospora* | |  | 1 | 0 | 1 |
| Actinobacteria | Nocardioidaceae | | *Aeromicrobium* | |  | 0 | 1 | 1 |
| Actinobacteria | Microbacteriaceae | | *Agromyces* | |  | 0 | 0 | 1 |
| Verrucomicrobia | Akkermansiaceae | | *Akkermansia* | |  | 1 | 0 | 1 |
| Bacteroidetes | Rikenellaceae | | *Alistipes* | | x | 1 | 1 | 1 |
| Bacteroidetes | Prevotellaceae | | *Alloprevotella* | | x | 1 | 1 | 1 |
| Actinobacteria | Microbacteriaceae | | *Amnibacterium* | |  | 1 | 0 | 0 |
| Proteobacteria | Succinivibrionaceae | | *Anaerobiospirillum* | | x | 1 | 1 | 1 |
| Firmicutes | Eubacteriaceae | | *Anaerofustis* | |  | 1 | 0 | 1 |
| Tenericutes | Anaeroplasmataceae | | *Anaeroplasma* | | x | 1 | 1 | 1 |
| Firmicutes | Erysipelotrichaceae | | *Anaerorhabdus_furcosa_group* | | x | 1 | 1 | 1 |
| Actinobacteria | Micrococcaceae | | *Arthrobacter* | | x | 1 | 1 | 1 |
| Firmicutes | Bacillaceae | | *Bacillus* | | x | 1 | 1 | 1 |
| Bacteroidetes | Bacteroidaceae | | *Bacteroides* | | x | 1 | 1 | 1 |
| Bacteroidetes | Barnesiellaceae | | *Barnesiella* | | x | 1 | 1 | 1 |
| Firmicutes | Lachnospiraceae | | *Blautia* | | x | 1 | 1 | 1 |
| Firmicutes | Paenibacillaceae | | *Brevibacillus* | | x | 1 | 1 | 1 |
| Firmicutes | Erysipelotrichaceae | | *Breznakia* | | x | 1 | 1 | 1 |
| Firmicutes | Ruminococcaceae | | *Butyricicoccus* | | x | 1 | 1 | 1 |
| Bacteroidetes | Marinifilaceae | | *Butyricimonas* | |  | 0 | 0 | 0 |
| Epsilonbacteraeota | Campylobacteraceae | | *Campylobacter* | | x | 1 | 1 | 1 |
| Firmicutes | Erysipelotrichaceae | | *Catenisphaera* | | x | 1 | 1 | 1 |
| Actinobacteria | Cellulomonadaceae | | *Cellulomonas* | | x | 1 | 1 | 1 |
| Actinobacteria | Promicromonosporaceae | | *Cellulosimicrobium* | |  | 0 | 1 | 1 |
| Firmicutes | Christensenellaceae | | *Christensenellaceae* | | x | 1 | 1 | 1 |
| Verrucomicrobia | Chthoniobacteraceae | | *Chthoniobacter* | |  | 1 | 0 | 1 |
| Firmicutes | Clostridiaceae | | *Clostridium* | | x | 1 | 1 | 1 |
| Actinobacteria | Coriobacteriaceae | | *Collinsella* | | x | 1 | 1 | 1 |
| Actinobacteria | Solirubrobacteraceae | | *Conexibacter* | | x | 1 | 1 | 1 |
| Actinobacteria | Corynebacteriaceae | | *Corynebacterium* | | x | 1 | 1 | 1 |
| Proteobacteria | Acetobacteraceae | | *Craurococcus* | |  | 0 | 0 | 1 |
| Actinobacteria | Microbacteriaceae | | *Curtobacterium* | |  | 1 | 0 | 1 |
| Firmicutes | Defluviitaleaceae | | *Defluviitaleaceae* | | x | 1 | 1 | 1 |
| Actinobacteria | Demequinaceae | | *Demequina* | |  | 1 | 0 | 0 |
| Firmicutes | Veillonellaceae | | *Dendrosporobacter* | | x | 1 | 1 | 1 |
| Proteobacteria | Devosiaceae | | *Devosia* | |  | 1 | 1 | 0 |
| Firmicutes | Erysipelotrichaceae | | *Dielma* | | x | 1 | 1 | 1 |
| Firmicutes | Lachnospiraceae | | *Eisenbergiella* | |  | 0 | 1 | 1 |
| Firmicutes | Enterococcaceae | | *Enterococcus* | | x | 1 | 1 | 1 |
| Actinobacteria | Eggerthellaceae | | *Enterorhabdus* | | x | 1 | 1 | 1 |
| Firmicutes | Erysipelotrichaceae | | *Erysipelatoclostridium* | | x | 1 | 1 | 1 |
| Firmicutes | Erysipelotrichaceae | | *Erysipelotrichaceae* | | x | 1 | 1 | 1 |
| Proteobacteria | Enterobacteriaceae | | *Escherichia_Shigella* | | x | 1 | 1 | 1 |
| Firmicutes | Eubacteriaceae | | *Eubacterium* | |  | 1 | 1 | 0 |
| Firmicutes | Clostridiales | | *Eubacterium_brachy_group* | | x | 1 | 1 | 1 |
| Firmicutes | Ruminococcaceae | | *Eubacterium_coprostanoligenes_group* | | x | 1 | 1 | 1 |
| Firmicutes | Eubacteriaceae | | *Eubacterium_nodatum_group* | | x | 1 | 1 | 1 |
| Firmicutes | Erysipelotrichaceae | | *Faecalitalea* | | x | 1 | 1 | 1 |
| Firmicutes | Ruminococcaceae | | *Flavonifractor* | | x | 1 | 1 | 1 |
| Fusobacteria | Fusobacteriaceae | | *Fusobacterium* | | x | 1 | 1 | 1 |
| Planctomycetes | Gemmataceae | | *Gemmata* | |  | 1 | 0 | 0 |
| Actinobacteria | Geodermatophilaceae | | *Geodermatophilus* | | x | 1 | 1 | 1 |
| Actinobacteria | Nocardiaceae | | *Gordonia* | |  | 1 | 0 | 0 |
| Epsilonbacteraeota | Helicobacteraceae | | *Helicobacter* | | x | 1 | 1 | 1 |
| Firmicutes | Ruminococcaceae | | *Intestinimonas* | |  | 0 | 1 | 1 |
| Actinobacteria | Promicromonosporaceae | | *Isoptericola* | | x | 1 | 1 | 1 |
| Actinobacteria | Kineosporiaceae | | *Kineococcus* | | x | 1 | 1 | 1 |
| Actinobacteria | Micrococcaceae | | *Kocuria* | |  | 1 | 1 | 0 |
| Firmicutes | Lachnospiraceae | | *Lachnoclostridium* | | x | 1 | 1 | 1 |
| Firmicutes | Lachnospiraceae | | *Lachnospiraceae* | | x | 1 | 1 | 1 |
| Firmicutes | Lactobacillaceae | | *Lactobacillus* | |  | 1 | 0 | 1 |
| Firmicutes | Streptococcaceae | | *Lactococcus* | | x | 1 | 1 | 1 |
| Firmicutes | Planococcaceae | | *Lysinibacillus* | |  | 1 | 1 | 0 |
| Firmicutes | Lachnospiraceae | | *Marvinbryantia* | | x | 1 | 1 | 1 |
| Proteobacteria | Beijerinckiaceae | | *Methylobacterium* | | x | 1 | 1 | 1 |
| Actinobacteria | Micromonosporaceae | | *Micromonospora* | | x | 1 | 1 | 1 |
| Proteobacteria | Beijerinckiaceae | | *Microvirga* | |  | 1 | 0 | 0 |
| Deferribacteres | Deferribacteraceae | | *Mucispirillum* | | x | 1 | 1 | 1 |
| Actinobacteria | Mycobacteriaceae | | *Mycobacterium* | | x | 1 | 1 | 1 |
| Tenericutes | Mycoplasmataceae | | *Mycoplasma* | |  | 0 | 0 | 0 |
| Firmicutes | Ruminococcaceae | | *Negativibacillus* | | x | 1 | 1 | 1 |
| Actinobacteria | Nocardioidaceae | | *Nocardioides* | | x | 1 | 1 | 1 |
| Proteobacteria | Rhizobiaceae | | *Ochrobactrum* | | x | 1 | 1 | 1 |
| Bacteroidetes | Marinifilaceae | | *Odoribacter* | | x | 1 | 1 | 1 |
| Firmicutes | Ruminococcaceae | | *Oscillibacter* | | x | 1 | 1 | 1 |
| Firmicutes | Paenibacillaceae | | *Paenibacillus* | | x | 1 | 1 | 1 |
| Firmicutes | Peptostreptococcaceae | | *Paeniclostridium* | | x | 1 | 1 | 1 |
| Bacteroidetes | Paludibacteraceae | | *Paludibacter* | | x | 1 | 1 | 1 |
| Bacteroidetes | Tannerellaceae | | *Parabacteroides* | | x | 1 | 1 | 1 |
| Proteobacteria | Rhodobacteraceae | | *Paracoccus* | |  | 1 | 1 | 0 |
| Bacteroidetes | Prevotellaceae | | *Paraprevotella* | | x | 1 | 1 | 1 |
| Proteobacteria | Burkholderiaceae | | *Parasutterella* | |  | 0 | 0 | 0 |
| Firmicutes | Lactobacillaceae | | *Pediococcus* | | x | 1 | 1 | 1 |
| Firmicutes | Peptococcaceae | | *Pelotomaculum* | |  | 0 | 1 | 1 |
| Firmicutes | Peptostreptococcaceae | | *Peptoclostridium* | |  | 1 | 0 | 1 |
| Firmicutes | Peptococcaceae | | *Peptococcus* | | x | 1 | 1 | 1 |
| Firmicutes | Acidaminococcaceae | | *Phascolarctobacterium* | | x | 1 | 1 | 1 |
| Bacteroidetes | Prevotellaceae | | *Prevotellaceae* | | x | 1 | 1 | 1 |
| Actinobacteria | Coriobacteriales | | *Raoultibacter* | | x | 1 | 1 | 1 |
| Proteobacteria | Burkholderiaceae | | *Rhizobium* | |  | 1 | 1 | 0 |
| Actinobacteria | Nocardiaceae | | *Rhodococcus* | | x | 1 | 1 | 1 |
| Bacteroidetes | Rikenellaceae | | *Rikenella* | | x | 1 | 1 | 1 |
| Bacteroidetes | Rikenellaceae | | *Rikenellaceae* | | x | 1 | 1 | 1 |
| Firmicutes | Peptostreptococcaceae | | *Romboutsia* | | x | 1 | 1 | 1 |
| Firmicutes | Lachnospiraceae | | *Roseburia* | | x | 1 | 1 | 1 |
| Proteobacteria | Acetobacteraceae | | *Roseomonas* | | x | 1 | 1 | 1 |
| Actinobacteria | Rubrobacteriaceae | | *Rubrobacter* | |  | 1 | 0 | 1 |
| Firmicutes | Ruminococcaceae | | *Ruminiclostridium* | | x | 1 | 1 | 1 |
| Firmicutes | Ruminococcaceae | | *Ruminococcaceae* | | x | 1 | 1 | 1 |
| Firmicutes | Ruminococcaceae | | *Ruminococcus* | | x | 1 | 1 | 1 |
| Firmicutes | Lachnospiraceae | | *Ruminococcus_gauvreauii_group* | | x | 1 | 1 | 1 |
| Firmicutes | Lachnospiraceae | | *Ruminococcus_torques_group* | | x | 1 | 1 | 1 |
| Firmicutes | Lachnospiraceae | | *Sellimonas* | | x | 1 | 1 | 1 |
| Planctomycetes | Isosphaeraceae | | *Singulisphaera* | | x | 1 | 1 | 1 |
| Actinobacteria | Eggerthellaceae | | *Slackia* | | x | 1 | 1 | 1 |
| Actinobacteria | Solirubrobacteraceae | | *Solirubrobacter* | |  | 1 | 0 | 1 |
| Proteobacteria | Sphingomonadaceae | | *Sphingomonas* | |  | 1 | 0 | 1 |
| Firmicutes | Streptococcaceae | | *Streptococcus* | | x | 1 | 1 | 1 |
| Actinobacteria | Streptomycetaceae | | *Streptomyces* | | x | 1 | 1 | 1 |
| Proteobacteria | Burkholderiaceae | | *Sutterella* | |  | 0 | 0 | 0 |
| Firmicutes | Bacillaceae | | *Terribacillus* | |  | 1 | 0 | 0 |
| Spirochaetes | Spirochaetaceae | | *Treponema* | | x | 1 | 1 | 1 |
| Firmicutes | Alicyclobacillaceae | | *Tumebacillus* | |  | 1 | 0 | 1 |
| Firmicutes | Erysipelotrichaceae | | *Turicibacter* | | x | 1 | 1 | 1 |
| Firmicutes | Lachnospiraceae | | *Tyzzerella* | | x | 1 | 1 | 1 |
| Firmicutes | Leuconostocaceae | | *Weissella* | | x | 1 | 1 | 1 |
|  |  | | **Totals:** | | 83 | 108 | 95 | 102 |
