# Supplementary material for: Impact of animal socioecology on gut microbial communities: Insights from wild meerkats in the Kalahari
Source: J Anim Ecol. 2025 Oct 30;94(12):2687–703. doi: 10.1111/1365-2656.70168 (PMC12673242; doi:10.1111/1365-2656.70168)
Supplement: Supplementary file 6 — Table S3. Multivariate MR‐QAP to examine the effects of group membership (‘same’ vs. ‘different’ groups) and kinship (Wang's relatedness coefficient) on the degree of Jaccard pairwise β‐diversity similarity in bacterial community composition (ASV abundance and diversity). Period of study was included as a control variable. [file JANE-94-2687-s004.docx]

**Supporting Table 3:** Multivariate MR-QAP to examine the effects of group membership (‘same’ versus ‘different’ groups) and kinship (Wang’s relatedness coefficient) on the degree of Jaccard pairwise *β*-diversity similarity in bacterial community composition (ASV abundance and diversity). Period of study was included as a control variable.

| Predictor | B | *Df* | F | p (F) |
| --- | --- | --- | --- | --- |
| Intercept | 3.6*** | 9312 | 72,15 | <0.001*** |
| Kinship | 0.03 |  |  |  |
| Group membership | 0.26** |  |  |  |
| Study period | 0.26*** |  |  |  |

***p < 0.001; **p < 0.01
