# Supplementary material for: Impact of animal socioecology on gut microbial communities: Insights from wild meerkats in the Kalahari
Source: J Anim Ecol. 2025 Oct 30;94(12):2687–703. doi: 10.1111/1365-2656.70168 (PMC12673242; doi:10.1111/1365-2656.70168)
Supplement: Supplementary file 7 — Table S4. Univariate MR‐QAPs to examine within‐group effects of kinship (Wang's relatedness coefficient) on the degree of Jaccard pairwise β‐diversity similarity in bacterial community composition (ASV abundance and diversity). [file JANE-94-2687-s006.docx]

**Supporting Table 4:** Univariate MR-QAPs to examine within-group effects of kinship (Wang's relatedness coefficient) on the degree of Jaccard pairwise *β*-diversity similarity in bacterial community composition (ASV abundance and diversity).

| Study period & group | *β* (from each MR-QAP) |
| --- | --- |
| 2006-2007: Group I | 0.26** |
| 2006-2007: Group II | 0.13* |
| 2006-2007: Group III | 0.11(*) |
| 2006-2007: Group IV | 0.03 |
| 2014-2015: Group V | 0.59** |
| 2014-2015: Group VI | 0.10(*) |
| 2016-2017: Group VII | -0.06 |
| 2016-2017: Group VIII | -0.05 |

**p < 0.01; *p < 0.05; (*) 0.05 < p < 0.1
