# Supplementary material for: Impact of animal socioecology on gut microbial communities: Insights from wild meerkats in the Kalahari
Source: J Anim Ecol. 2025 Oct 30;94(12):2687–703. doi: 10.1111/1365-2656.70168 (PMC12673242; doi:10.1111/1365-2656.70168)
Supplement: Supplementary file 8 — Table S5. Parameter estimates from a generalised linear mixed model (GLMM) used to evaluate the fit of CSR2 values (outcome variables) of four joint‐species distribution models (JSDMs). [file JANE-94-2687-s009.docx]

**Supporting Table 5:** Parameter estimates from a generalized linear mixed model (GLMM) used to evaluate the fit of CSR^2^ values (outcome variables) of four joint-species distribution models (JSDMs). The full model incorporated all covariates, while the three covariate-specific models included only host social, host biological, and environmental covariates, respectively.

| **Estimate** | **B** | **SE** | **z** | **P** |
| --- | --- | --- | --- | --- |
| (Intercept) | 1.03 | 0.10 | 9.85 | <0.001 |
| Full model vs ‘host social covariates only’ model | 0.32 | 0.04 | 7.08 | <0.001 |
| Full model vs ‘host biological covariates only’ model | 0.36 | 0.05 | 7.78 | <0.001 |
| Full model vs ‘environmental covariates only’ model | 0.11 | 0.04 | 2.54 | 0.011 |
