# Supplementary material for: Impact of animal socioecology on gut microbial communities: Insights from wild meerkats in the Kalahari
Source: J Anim Ecol. 2025 Oct 30;94(12):2687–703. doi: 10.1111/1365-2656.70168 (PMC12673242; doi:10.1111/1365-2656.70168)
Supplement: Supplementary file 9 — Table S6. Parameter estimates from GLMMs used to compare the mean effect sizes of covariates, on the relative abundance of bacterial ASVs. [file JANE-94-2687-s003.docx]

**Supporting Table 6:** Parameter estimates from GLMMs used to compare the mean effect sizes of covariates, on the relative abundance of bacterial ASVs. The outcome variables were the standardised *β*-coefficient values extracted from the JSDM output. Predictors included ‘covariate type’ as a main effect, and ‘covariate category’ and ‘bacterial ASV’ as random effects. Panels A – C show comparisons among social, biological, and environmental covariates respectively. Panels D – F show comparisons between each social covariate and all the biological and environmental covariates. All indications of significant differences are based on critical p values corrected for multiple comparisons (p_corr_ values specified for each model below).

| **Covariates compared** | ***β*** | **Std. Error** | **z** | **p** |
| --- | --- | --- | --- | --- |
| 1. **Comparisons among social covariates (p_corr_ = 0.017):** | | | | |
| Group membership vs Recent immigration | 0.98 | 0.11 | 8.87 | <0.001* |
| Group membership vs Dominance status | 0.72 | 0.11 | 6.67 | <0.001* |
| Recent immigration vs Dominance status | -0.22 | 0.12 | -1.90 | 0.06 |
| 1. **Comparisons among biological (demographic and health-related) covariates (p_corr_ = 0.008):** | | | | |
| Age vs Sex | 2.39 | 0.11 | 22.24 | <0.001* |
| Age vs Body condition | 1.44 | 0.10 | 15.00 | <0.001* |
| Age vs Disease status | 1.93 | 0.10 | 18.96 | <0.001* |
| Sex vs Body condition | -0.95 | 0.12 | -8.22 | <0.001* |
| Sex vs Disease status | -0.46 | 0.12 | -3.82 | <0.001* |
| Body condition vs Disease status | 0.46 | 0.11 | 4.13 | <0.001* |
| 1. **Comparisons among environmental (climatic and temporal) covariates (p_corr_ = 0.008):** | | | | |
| Temperature vs Rainfall | -0.09 | 0.10 | -0.84 | 0.40 |
| Temperature vs Time since foraging | 0.31 | 0.11 | 2.83 | 0.005* |
| Temperature vs Hours after sunrise | -1.32 | 0.10 | -13.88 | <0.001* |
| Rainfall vs Time since foraging | 0.39 | 0.11 | 3.65 | <0.001* |
| Rainfall vs Hours after sunrise | -1.24 | 0.09 | -13.03 | <0.001* |
| Time since foraging vs Hours after sunrise | -1.63 | 0.10 | -16.39 | <0.001* |
| 1. **Social: Group membership compared to biological & environmental covariates (p_corr_ = 0.006):** | | | | |
| Group membership vs Host biological (demographic): age | -1.17 | 0.09 | -12.69 | <0.001* |
| Group membership vs Host biological (demographic): sex | 1.22 | 0.11 | 10.72 | <0.001* |
| Group membership vs Host biological (health-related): body condition | 0.27 | 0.10 | 2.56 | 0.005* |
| Group membership vs Host biological (health-related): disease status` | 0.76 | 0.11 | 6.99 | <0.001* |
| Group membership vs Environmental (climate): temperature | 0.17 | 0.10 | 1.69 | 0.09 |
| Group membership vs Environmental (climate): rainfall | 0.09 | 0.10 | 0.84 | 0.40 |
| Group membership vs Environmental (temporal): time since foraging | 0.48 | 0.11 | 4.52 | <0.001* |
| Group membership vs Environmental (temporal): hours after sunrise | -1.15 | 0.09 | -12.33 | <0.001* |
| **(E) Social: Recent immigration compared to biological & environmental covariates (p_corr_ = 0.006):** | | | | |
| Recent immigration vs Host biological (demographic): age | -2.16 | 0.10 | -20.67 | <0.001* |
| Recent immigration vs Host biological (demographic): sex | 0.23 | 0.12 | 1.92 | 0.005* |
| Recent immigration vs Host biological (health-related): body condition | -0.72 | 0.11 | -6.33 | <0.001* |
| Recent immigration vs Host biological (health-related): disease status | -0.22 | 0.12 | -1.90 | 0.05 |
| Recent immigration vs Environmental (climate): temperature | -0.81 | 0.11 | -7.21 | <0.001* |
| Recent immigration vs Environmental (climate): rainfall | -0.90 | 0.11 | -8.00 | <0.001* |
| Recent immigration vs Environmental (temporal): time since foraging | -0.51 | 0.12 | -4.40 | <0.001* |
| Recent immigration vs Environmental (temporal): hours after sunrise | -2.14 | 0.11 | -20.21 | <0.001* |
| **(F) Social: Dominance status compared to biological & environmental covariates (p_corr_ = 0.006):** | | | | |
| Dominance status vs Host biological (demographic): age | -1.90 | 0.10 | -18.69 | <0.001* |
| Dominance status vs Host biological (demographic): sex | 0.49 | 0.12 | 4.14 | <0.001* |
| Dominance status vs Host biological (health-related): body condition | -0.46 | 0.11 | -4.13 | <0.001* |
| Dominance status vs Host biological (health-related): disease status | 0.04 | 0.12 | 0.32 | 0.75 |
| Dominance status vs Environmental (climate): temperature | -0.55 | 0.11 | -5.01 | <0.001* |
| Dominance status vs Environmental (climate): rainfall | -0.64 | 0.11 | -5.82 | <0.001* |
| Dominance status vs Environmental (temporal): time since foraging | -0.25 | 0.11 | -2.18 | 0.003* |
| Dominance status vs Environmental (temporal): hours after sunrise | -1.88 | 0.10 | -18.26 | <0.001* |

*Observed p-value is less than the critical p-value corrected for multiple comparisons (p_corr_).
